# Glioblastoma, *IDH*-Wild Type With *FGFR3-TACC3* Fusion: When Morphology May Reliably Predict the Molecular Profile of a Tumor. A Case Report and Literature Review

**DOI:** 10.3389/fneur.2022.823015

**Published:** 2022-02-09

**Authors:** Giuseppe Broggi, Eliana Piombino, Roberto Altieri, Chiara Romano, Francesco Certo, Giuseppe Maria Vincenzo Barbagallo, Paolo Vigneri, Dario Condorelli, Lorenzo Colarossi, Cristina Colarossi, Gaetano Magro, Elena Tirrò

**Affiliations:** ^1^Department of Medical and Surgical Sciences and Advanced Technologies “G.F. Ingrassia”, Anatomic Pathology, University of Catania, Catania, Italy; ^2^Pathology Unit, Department of Experimental Oncology, Mediterranean Institute of Oncology, Catania, Italy; ^3^Department of Medical and Surgical Sciences and Advanced Technologies “G.F. Ingrassia”, Neurological Surgery, Policlinico “G. Rodolico-San Marco” University Hospital, University of Catania, Catania, Italy; ^4^Department of Clinical and Experimental Medicine, University of Catania, Catania, Italy; ^5^Center of Experimental Oncology and Hematology, A.O.U. Policlinico “G. Rodolico-San Marco”, Catania, Italy; ^6^Department of Medical and Surgical Sciences and Advanced Technologies “GF Ingrassia”, University of Catania, Catania, Italy; ^7^Department of Surgical, Oncological and Stomatological Sciences, University of Palermo, Palermo, Italy

**Keywords:** FGFR3-TACC3 fusion, glioblastoma, unusual morphological features, molecular biology, diagnosis, IDH-wildtype, high-grade glioma

## Abstract

It has been reported that in-frame *FGFR3-TACC3* fusions confer to glioblastomas, *IDH*-wild type (GBMs, *IDH*wt) some unusual morphologic features, including monomorphous rounded cells with ovoid nuclei, nuclear palisading, endocrinoid network of “chicken-wire” vessels, microcalcifications and desmoplastic stroma, whose observation may predict the molecular profile of the tumor. We herein present a case of recurrent GBMs, *IDH*wt, exhibiting some of the above-mentioned morphological features and a molecularly-proven *FGFR3-TACC3* fusion. A 56-year-old man presented to our hospital for a recurrent GBM, *IDH*wt, surgically treated at another center. Histologically, the tumor, in addition to the conventional GBM morphology, exhibited the following peculiar morphologic features: (1) monomorphous neoplastic cells with rounded nuclei and scant pale cytoplasm; (2) thin capillary-like vessels with “chicken-wire” pattern; (3) nuclear palisading; (4) formation of vague perivascular pseudorosettes; (5) spindled tumor cells embedded in a loose, myxoid background. Based on this unusual morphology, molecular analyses were performed and an *FGFR3* exon17-*TACC3* exon 10 fusion was found. The present case contributes to widening the morphologic spectrum of *FGFR3-TACC3*-fused GBM, IDHwt and emphasizes that pathologists, in the presence of a GBM, *IDH*wt with unconventional morphology, should promptly search for this fusion gene.

## Introduction

Adult glioblastomas, *IDH*-wild type comprise a molecularly and histopathologically heterogeneous spectrum of neoplasms, characterized by poor prognosis and frequent resistance to the conventional radio-chemotherapy treatments (1–3). According to the cIMPACT-NOW criteria (4), the molecular diagnosis of glioblastomas, *IDH*-wild type (GBMs, *IDH*wt) is essentially based on the presence of at least one of the following alterations in the context of an adult diffuse astrocytic neoplasm, *IDH*-wt: i) combined *7p* gain and *10q* loss, ii) *epidermal growth factor receptor* (*EGFR*) amplification, and iii) *telomerase reverse transcriptase* (*TERT*) promoter mutation (5, 6). GBM, *IDH*wt also shows a wide morphological spectrum, and some histopathologic variants exhibit additional molecular alterations with potential therapeutic implications (7). Genomic profiling studies revealed that GBMs show an extensive molecular heterogeneity and about 30–50% of malignant gliomas harbor targetable gene fusion mainly involving *EGFR*, neurotrophic tyrosine receptor kinase (*NTRK*), and fibroblast growth factor receptor (*FGFR*) genes (8). In the past, *fibroblast growth factor receptor 3* (*FGFR3*)-*transforming acidic coiled-coil 3* (*TACC3*) gene fusion was identified as a rare molecular feature in grade 1 to 4 adult diffuse gliomas lacking *IDH1/2* mutations but always carrying *TERT* promoter mutations or *CDKN2A* loss in about 75% of cases (9, 10). The *FGFR3-TACC3* gene fusion acts as an oncogene, encoding a protein, located on mitotic spindle poles, with constitutive kinase function, that causes a loss of the normal chromosomal segregation and stimulates aneuploidy (11). The identification of the oncogenic *FGFR3-TACC3* fusion highlighted the possibility of identifying a subset of diffuse glioma patients potentially responsive to targeted therapy with *FGFR* kinase inhibitors (12, 13). In the last few years, Bielle et al. have described a series of 30 adult high-grade diffuse gliomas, harboring an in-frame *FGFR3-TACC3* fusion and exhibiting the conventional molecular alterations of GBMs, *IDH*wt, but peculiar histopathologic features; interestingly, the following unusual morphological features were found: “*monomorphous ovoid nuclei, nuclear palisading, and thin parallel cytoplasmic processes, an endocrinoid network of thin capillaries) associated with frequent microcalcifications and desmoplasia”* (14). Since then, additional cases with the co-occurrence of *FGFR3-TACC3* fusion and the above-mentioned histopathologic features have been reported in the literature (15), raising the question of whether this unusual morphology may predict the presence of this equally rare molecular finding.

We herein report a case of a 56-year-old male patient affected by a recurrent GBM, *IDH*wt, showing both an unconventional morphology and a molecularly-proven *FGFR3-TACC3* gene fusion. A critical review of the literature that emphasizes the potential association between morphology and molecular status of this GBM subtype is also included.

## Case Presentation

**Figure 1:**
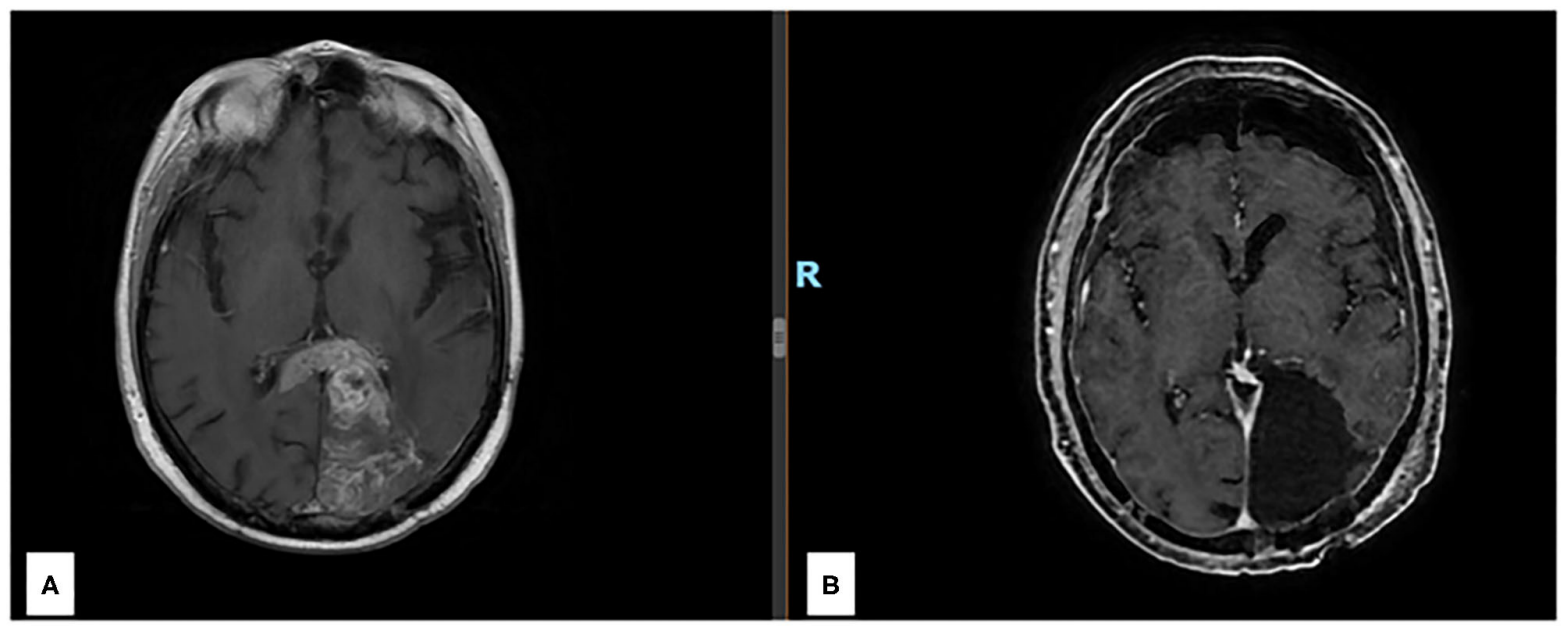
**(A)** Preoperative axial section of a T1 w MRI after gadolinium injection revealing a left parieto-occipital recurrent lesion with infiltration of the splenium of the corpus callosum. **(B)** Postoperative axial section of a T1 w MRI after gadolinium injection revealing the complete resection of the enhancing nodule.

A 56-year-old man was admitted to our hospital on March 2021 for the recurrence of a GBM, *IDH*wt, which had been surgically treated with a subtotal resection at another center in October 2017. After Stupp regimen and some months of wellness, he developed aphasia and confusion. Brain MRI showed a left parieto-occipital mass with infiltration of the splenium of the corpus callosum (Figure 1A) and a gross total resection with a good clinical result was surgically achieved (Figure 1B).

**Figure 2:**
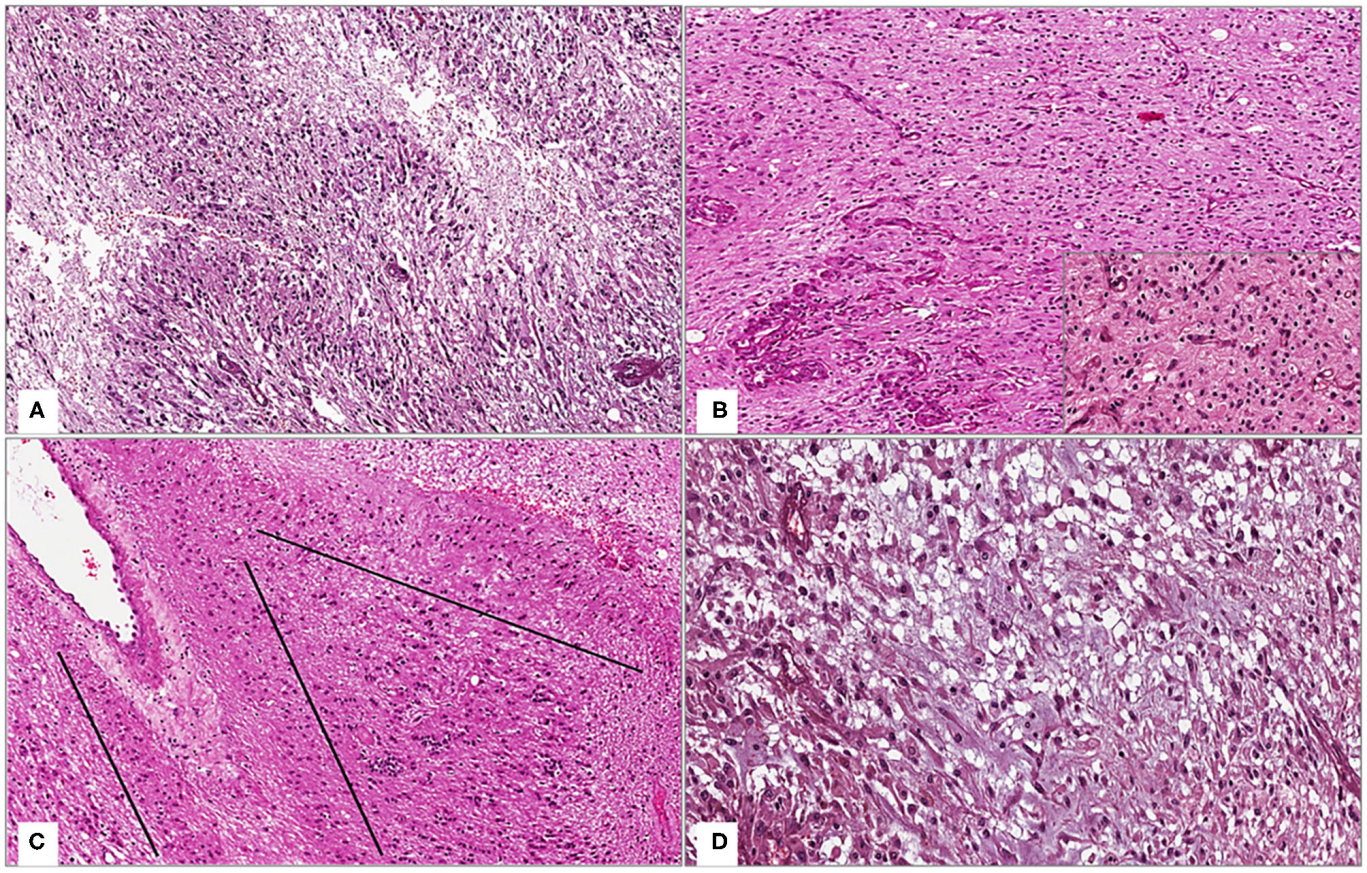
**(A)** Low magnification showing the conventional morphology of WHO grade IV glioblastoma, IDH-wild type: a moderately cellular astrocytic tumor with foci of pseudopalisading necrosis (hematoxylin and eosin; original magnification 150x); **(B)** Tumor exhibits, as an unusual morphologic feature, more bland-looking areas composed of monomorphous round-shaped cells and thin capillary-like vessels with “chicken-wire” pattern, arranged in an endocrinoid pattern (insert); these features are also found close to foci of microvascular proliferation [hematoxylin and eosin; original magnifications 150x and 300x (insert)]; **(C)** Tumor areas with nuclear palisading (lines) are seen (hematoxylin and eosin; original magnification 150x); **(D)** Spindled neoplastic cells set in a loose, myxoid background, imparting to the tumor a focal “tissue culture-like” morphology (hematoxylin and eosin; original magnification 300x).

Histologically, the tumor was composed of spindled to rounded astrocytic cells that showed an infiltrating growth pattern and high-grade features, such as hypercellularity, high mitotic index (nine mitoses per 10 high-power fields), foci of microvascular proliferation, and pseudopalisading necrosis (Figure 2A). Interestingly, the tumor also exhibited some unusual morphological features (Figures 2B–D): i) presence of monomorphous ovoid cells with rounded nuclei and sometimes scant pale cytoplasm; ii) numerous thin capillary-like vessels with “chicken-wire” pattern, arranged in an endocrinoid pattern; iii) nuclear palisading; iv) focal perivascular arrangement of neoplastic cells, resulting in the formation of vague perivascular pseudorosettes; v) spindled neoplastic cells embedded in a loose, myxoid background, producing a “tissue culture-like” appearance. Neither microcalcifications, desmoplastic stroma, nor histologic signs of previous treatments were seen. The above-mentioned unusual morphological features were found both distant and in close proximity to tumor areas containing foci of necrosis and microvascular proliferation (Figure 2B). Neoplastic cells were diffusely stained with GFAP and OLIG-2. No immunoexpression of IDH1 R132H, H3K27M, H3G34M, and CD34 was found. Nuclear expressions of ATRX and H3K27me3 were retained; <10% of the neoplastic cells were stained with p53 and the Ki-67 proliferation rate was about 10%. Based on both morphological and immunohistochemical features, a diagnosis of recurrent “*WHO grade 4 glioblastomas, IDH-wild type”* was rendered.

**Figure 3:**
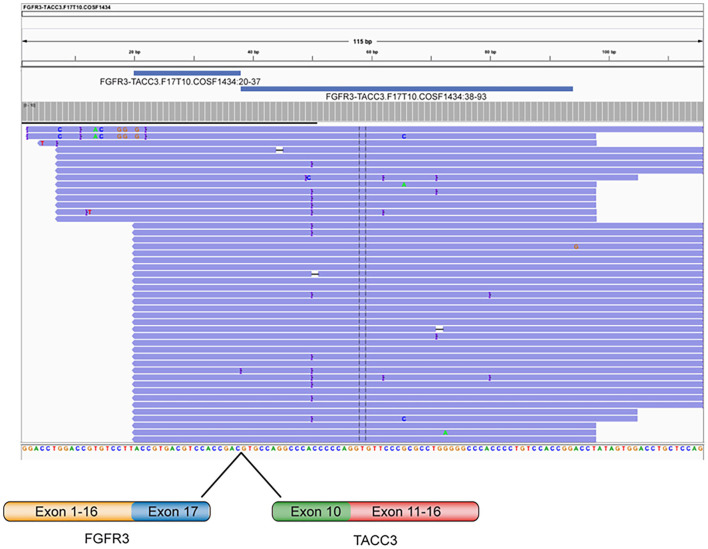
Representation of the *FGFR3-TACC3* fusion gene identified by Next Generation Sequencing in a GBM *IDH*wt patient. Visualization of RNA sequencing reads supports the fusion junction between *FGFR3* exon 17 and *TACC3* exon 10.

Subsequently, because of the unusual morphology encountered, next-generation sequencing (NGS) was chosen to identify further molecular alterations. NGS was performed using a custom panel for the identification of point mutations, INDEL and copy number variations (Glio-panel DNA), and a custom panel for the detection of gene fusions (Glio-panel RNA). The RNA sequencing of recurrent GBM revealed the presence of *FGFR3* exon17-*TACC3* exon 10 (Catalog of Somatic Mutations in Cancer mutation identifier COSF1434) fusion (Figure 3). Moreover, NGS sequencing identified the presence of the most common mutations associated with *FGFR3-TACC3* fusion in GBM, *IDH*wt: the pathogenic deletion on the *PTEN* gene (p.Trp111Ter) and *TERT* c.C228T promoter mutation (16). Furthermore, chromosome 10q loss without chromosome seven gain was detected, while no *EGFR, MDM2*, and *CDK4* amplification nor *CDKN2A* homozygous deletion were found in the analyzed sample.

## Discussion

*FGFR3-TACC3* fusions are oncogenic drivers that were first reported in GBMs and bladder urothelial carcinomas (17); in more detail, this unusual fusion was first detected on a series of 97 GBM cases, two of which harbored the *FGFR3-TACC3* fusion (18). Subsequently, larger molecular studies on 584 GBMs and 211 lower-grade diffuse gliomas reported 17 GBMs and three lower-grade gliomas with FGFR3-TACC3 fusions (11). Based on other studies reported in the literature, it is estimated that only a small percentage of GBMs (1–8%) harbor this gene fusion and the incidence decreases further if grade 2 and 3 diffuse gliomas are also considered (17). *FGFR3-TACC3* fusions, although less frequently reported than *FGFR2* and *BRAF* alterations, have also been identified in cases of “polymorphous low-grade neuro-epithelial tumor of the young” (PLNTY) (19).

In 2017 Bielle et al. reported a series of 30 patients affected by *FGFR3-TACC3*-fused adult gliomas (age range: 42–87 years), which exhibited some unusual morphological features, combined with microcalcifications and desmoplasia; their cohort included 25 cases of GBMs, *IDH*wt, one case of gliosarcoma, *IDH*wt, one case of GBM, not otherwise specified, two cases of diffuse astrocytomas “with molecular features of GBM” (7p gain, 10q loss, and *TERT* promoter mutation) and one case of histological grade 2 diffuse astrocytoma, *IDH*wt with no additional molecular analyses available (14). Furthermore, 73% of these cases showed some recurrent unusual morphological features, including monomorphous ovoid nuclei, endocrinoid network of capillary vessels, vague formation of perivascular pseudorosettes, nuclear palisading, microcalcifications, and desmoplastic stroma. The presence of this unusual morphology in GBM cases was restricted to areas that lacked necrosis and/or microvascular proliferation and extravascular immunohistochemical staining for CD34 was found in about 50% of cases. These tumors molecularly showed, in addition to the *FGFR3-TACC3* fusion, the conventional GBM, *IDH*wt features (absence of *IDH1/2, ATRX* and *TP53* mutations, 7p gain, 10q loss, and *TERT* promoter mutations), except for *EGFR* amplification (0/29), combined with a higher incidence of *CDKN2A* homozygous deletions.

The study of Gilani et al. recently described the histopathologic features of six adult GBMs, *IDH*wt with *FGFR3-TACC3* fusion and lack of *EGFR* amplification, confirming the presence of the above-mentioned unusual morphologic features, variably combined, in five out of six cases. The remaining case, despite harboring the *FGFR3-TACC3* fusion, exhibited a different morphology from that previously published, characterized by less “bland-looking” cellularity and more striking nuclear atypia (15). Despite being aware that the detection of monomorphous ovoid cells with endocrinoid network of vessels, microcalcifications, and desmoplasia on a high-grade glioma, *IDH*wt might justify the search for *FGFR3-TACC3* fusions, the authors concluded that morphology alone could not predict the molecular status of these rare subsets of GBMs, as some *FGFR3-TACC3*-fused cases, that lacked these peculiar features, occurred, and, conversely, GBM cases, exhibiting this unusual morphology, lacked the *FGFR3-TACC3* fusion.

The present paper reports an additional case of a recurrent GBM, *IDH*wt, and *FGFR3-TACC3* fused with emphasis on the potential correlation between histopathology and molecular status. Histologically, our case showed tumor areas with conventional morphology of GBM, alternating with areas with some of the above-mentioned unusual morphological features. Compared to those cases reported in the literature, the present case showed, as an additional and previously unreported morphologic feature, a spindled neoplastic component, embedded in a loose, myxoid background, producing a “tissue culture-like” appearance. These particular histopathologic features were also found close to tumor areas with necrosis and foci of microvascular proliferation and led us to request a further molecular test for diagnostic confirmation and for the search of *FGFR3-TACC3* fusion, whose presence has not only a speculative but also a practical function as it identifies a subset of patients with a slightly better prognosis than those affected by conventional GBM, *IDH*wt and who could benefit from a targeted therapy with *FGFR* kinase inhibitors. As some of these uncommon morphologic features are shared with other brain tumors, they often represent diagnostic challenges: i) oligodendrogliomas, *IDH*-mutant, and 1p/19q codeleted often exhibit monomorphic rounded cells with pale cytoplasm and a “chicken-wire” vascular network; ii) ependymomas and angiocentric glioma characteristically show perivascular pseudorosettes; iii) glioneuronal tumors, in general, may exhibit extravascular positivity for CD34 and desmoplastic stroma (14). These histological findings in a diffuse glioma *IDH*-wildtype should prompt pathologists to consider *FGFR3-TACC3* fusion and look for additional genetic alterations that are required for the diagnosis of GBM, *IDH*wt. The treatment for patients with GBM includes combined radio and chemotherapy (20). Temozolomide (TMZ) is the standard chemotherapeutic used alone or in association with a DNA alkylating agent: however, chemoresistance and not well-characterized mechanisms involved in the development of tumors are the most common cause of therapy failure (21–25). Furthermore, for recurrent gliomas, standard-of-care treatments are not well defined; treatment is usually selected based on prior therapy, age, Karnofsky Performance Scale (KPS), MGMT promoter methylation status, and patterns of disease progression (26). Bevacizumab, an anti-vascular endothelial growth factor (VEGF) monoclonal antibody that has been introduced in the USA in 2009 as a treatment for recurrent high-grade gliomas, has become one of the first-choice therapies for recurrent GBMs, according to National Comprehensive Cancer Network (NCCN) guidelines. The combination of bevacizumab and chemotherapy represents an additional treatment option for these patients. However, when the standard therapeutic regimens lack efficacy, targeted therapies for patients with primary and recurrent GBMs are currently limited, and novel molecular biomarkers are needed to improve the development of personalized treatments.

Xu et al. reported that potentially targetable molecular alterations, mainly involving *NTRK, EGFR*, and *FGFR* genes, occurred in about 30 to 50% of GBMs (8). In more detail, while *NTRK* rearrangements are very rare, being found in <2% of GBM cases and consisting of fusions between *NTRK1* and other genes, such as *NFASC, BCAN, CHTOP*, and *ARHGEF2, EGFR* in-frame fusions are much more frequent (*EGFR-SEPT14* and *EGFR-PSPH* fusion genes were observed approximately in 4 and 2% of cases) and frequently lead to EGFR overexpression in GBM (8); however, all clinical trials with EGFR inhibitors did not demonstrate longer survival times in GBM patients so far, probably due to the inclusion of poorly homogeneous patient populations. Finally, these authors reported that about 1 to 8% of GBMs harbored potentially druggable *FGFR-TACC* rearrangements, being *FGFR3-TACC3* the gene fusion most frequently encountered in 5% of cases, followed by *FGFR1-TACC1* (8). Nowadays, tyrosine kinase fusion genes are an important class of oncogenes associated with different hematological and solid tumors (27), thus targeting gene fusion has been a promising therapeutic option in several types of cancer models (28–30). The growing therapeutic relevance of FGFR alterations, including fusions, in different cancer types, has greatly supported the development of a variety of tyrosine kinase inhibitors (TKIs) (31–33). Although these drugs exhibit good anticancer effects in many, their use in the treatment of brain malignancies is limited. Among the reasons for this is the presence of the blood-brain barrier that influences the delivery of drugs to the central nervous system as well as patient-to-patient variability.

The presence of the *FGFR3-TACC3* fusion gene certainly represents a further targetable mutation within the molecular heterogeneity typical of the majority of GBMs (34). The reassuring outcome of anti-FGFR inhibitors in different preclinical studies strengthened the rationale to employ FGFR tyrosine kinase inhibitors in GBM patients harboring the *FGFR3-TACC3* fusion gene (11, 18). Different clinical trials studies have been completed (NCT02824133 and NCT01975701) or are still recruiting GBM patients (NCT04424966 or NCT04547855) to test the efficacy of multi-targeted receptor tyrosine kinase inhibitors, such as Anlotinib, or selective FGFR1-3 inhibitors, such as Infigratinib, in relapsed or progressed GBM patients. In this regard, Wang et al. described a partial response (>17 months of follow-up) in a 44-year-old woman affected by recurrent GBM, *IDH*wt, that harbored simultaneously an *FGFR3-TACC3* fusion and *FGFR3* amplification, treated with Anlotinib 12 mg p.o. once every day plus oral TMZ chemotherapy (35). Interestingly, the authors speculated that the coexistence of two different *FGFR3* alterations *(FGFR3-TACC3* fusion and *FGFR3* amplification) in the same tumor could be the main reason for the significant efficacy of Anlotinib therapy and emphasized that tumors harboring *FGFR3-TACC3* rearrangements and/or *FGFR3* amplification should be selected for clinical trials featuring *FGFR* inhibitors (35).

## Conclusions

The present case highlights that neuropathologists should be aware that the presence of an unusual morphology may reliably predict a distinct molecular profile of GBM, *IDH*wt, and that, in the presence of the above-mentioned features, they must promptly consider a *FGFR3-TACC3* fusion. The spindle cell component embedded in a myxoid stroma, found in our case, contributes to expanding the spectrum of morphologic features that may predict the presence of *FGFR3-TACC3* fusions. To this end, the detection of a fusion gene using transcriptome sequencing may represent a novel approach (36). In conclusion, we strongly emphasize that the prompt identification of the combination between unusual morphology and presence of *FGFR3-TACC3* fusion has mainly the practical purpose of identifying a subset of patients with a slightly better outcome than those affected by conventional GBM, *IDH*wt, and for whom the use of personalized treatment with *FGFR* kinase inhibitors may be considered.

## Author Contributions

GB, EP, and ET: conceptualization. DC and ET: methodology. CR, PV, and ET: validation. GB and ET: formal analysis, writing–original draft preparation, writing–review, and editing. GB: investigation. EP, LC, and CC: resources. RA, FC, and GM: supervision. All authors have read and agreed to the published version of the manuscript.

## Funding

This study was partially funded by the Research plan of the University of Catania-Linea di intervento 2-entitled MultiDisciplinary RESEarch and Targeted Therapy for malignant GLIOmas (MD-RESETT-GLIO).

## Conflict of Interest

The authors declare that the research was conducted in the absence of any commercial or financial relationships that could be construed as a potential conflict of interest.

## Publisher's Note

All claims expressed in this article are solely those of the authors and do not necessarily represent those of their affiliated organizations, or those of the publisher, the editors and the reviewers. Any product that may be evaluated in this article, or claim that may be made by its manufacturer, is not guaranteed or endorsed by the publisher.
